# Identifying novel proteins for suicide attempt by integrating proteomes from brain and blood with genome-wide association data

**DOI:** 10.1038/s41386-024-01807-4

**Published:** 2024-02-05

**Authors:** Hao Zhao, Yifeng Liu, Xuening Zhang, Yuhua Liao, Huimin Zhang, Xue Han, Lan Guo, Beifang Fan, Wanxin Wang, Ciyong Lu

**Affiliations:** 1 Department of Medical Statistics and Epidemiology, School of Public Health, Sun Yat-sen University, Guangzhou, China; 2 Guangdong Provincial Key Laboratory of Food, Nutrition and Health, Sun Yat-sen University, Guangzhou, China; 3 Department of Psychiatry, Shenzhen Nanshan Center for Chronic Disease Control, Shenzhen, China; 4 Department of Epidemiology and Health Statistics, School of Public Health, Cheeloo College of Medicine, Shandong University, Jinan, China

**Keywords:** Human behaviour, Psychiatric disorders, Predictive markers, Proteomics, Behavioural genetics

## Abstract

Genome-wide association studies (GWASs) have identified risk loci for suicide attempt (SA), but deciphering how they confer risk for SA remains largely unknown. This study aims to identify the key proteins and gain insights into SA pathogenesis. We integrated data from the brain proteome (*N* = 376) and blood proteome (*N* = 35,559) and combined it with the largest SA GWAS summary statistics to date (*N* = 518,612). A comprehensive set of methods was employed, including Mendelian randomization (MR), Steiger filtering, Bayesian colocalization, proteome‑wide association studies (PWAS), transcript-levels, cell-type specificity, correlation, and protein-protein interaction (PPI) network analysis. Validation was performed using other protein datasets and the SA dataset from FinnGen study. We identified ten proteins (GLRX5, GMPPB, B3GALTL, FUCA2, TTLL12, ADCK1, MMAA, HIBADH, ACP1, DOC2A) associated with SA in brain proteomics. GLRX5, GMPPB, and FUCA2 showed strong colocalization evidence and were supported by PWAS and transcript-level analysis, and were predominantly expressed in glutamatergic neuronal cells. In blood proteomics, one significant protein (PEAR1) and three near-significant proteins (NDE1, EVA1C, B4GALT2) were identified, but lacked colocalization evidence. Moreover, despite the limited correlation between the same protein in brain and blood, the PPI network analysis provided new insights into the interaction between brain and blood in SA. Furthermore, GLRX5 was associated with the GSTP1, the target of Clozapine. The comprehensive analysis provides strong evidence supporting a causal association between three genetically determined brain proteins (GLRX5, GMPPB, and FUCA2) with SA. These findings offer valuable insights into SA’s underlying mechanisms and potential therapeutic approaches.

## Introduction

Suicide is a pressing global public health issue. According to the survey data of the World Health Organization (WHO), more than 800,000 people lose their lives to suicide every year, and the number of suicide attempt (SA) is several times higher than the number of suicide deaths [1, 2]. SA is defined as self-injurious behavior with the intent to die, and the lifetime prevalence of SA among adults globally is estimated to be around 0.5–5% [3]. This not only poses a risk of personal disability and reduced quality of life but also brings a heavy burden on families and society as a whole. SA is a complex result of the interaction of genetic, biological, psychological, environmental and social factors, making suicide a focus area of research [4]. Despite the many risk factors associated with SA, our understanding of the underlying mechanisms is still limited, and there is a lack of effective prevention strategies to reduce the prevalence of SA. The increase in large-scale genome-wide association studies (GWASs) over the past few years has greatly contributed to the identification of genetic variations associated with SA. The latest GWAS meta-analysis conducted by the International Suicide Genetics Consortium (ISGC) involved over 29,000 cases of SA or suicides from 18 cohorts worldwide [5]. The results revealed an estimated heritability of ~6.8% for SA, highlighting the important role of genetic factors in this complex behavior. However, deciphering the underlying biological processes responsible for most of these genetic effects remains challenging, hampering further understanding of the mechanisms behind SA and the discovery of biomarkers and the development of drug targets.

Proteins are among the most important biological molecules in cells, representing the main functional components of cells and biological processes and the ultimate products of gene expression [6]. With the widespread application of proteomic analysis techniques recently, a large number of protein quantitative trait loci (pQTLs) have been found in blood and brain [7–9]. These pQTLs, located near coding genes known as cis-pQTLs, are more likely to influence protein levels by directly affecting transcription or translation, which provides new possibilities for exploring the causal associations between proteins and SA from a genetic perspective. Proteins in the brain are closely related to the central nervous system and play important roles in regulating brain function, development, mental disorders, and SA [10, 11]. On the other hand, blood proteins are easier to obtain and detect, and they can reflect various physiological and pathological processes [12, 13]. Therefore, integrating data from brain and blood proteomics will provide new insights into the biological mechanisms of SA.

Mendelian randomization (MR) is a causal inference method that mimics the design of natural randomized controlled trials using genetic variation in single nucleotide polymorphisms (SNPs) as instrumental variables (IVs), which reducing measurement error and confounding, enabling reliable estimation of causal relationships between specific proteins and SA [14]. Steiger filtering is an extension of MR that calculates the variance explained by the exposure and outcome variables to determine the direction of possible causal relationships of proteins with SA [15]. Because linkage disequilibrium (LD) between different variants within a single genetic region may confuse MR results, Bayesian colocalization analysis was performed to calculate the probability of shared causal genetic variation between a specific protein and SA [16]. These methods have been applied to various diseases, such as depression [17], loneliness [18], post-traumatic stress disorder [19], Parkinson’s disease [20], and stroke [21]. However, the exploration of potential key proteins underlying SA remains limited.

In this study, we integrated brain and blood proteomic data with large-scale GWAS summary statistics from 14 European cohorts on SA. We employed various methods such as MR, Steiger filtering, and Bayesian colocalization to reveal causal relationships between specific proteins and SA. To validate the robustness of our findings, we conducted repeatability MR analysis using other protein datasets and the SA dataset from FinnGen study, and extension analysis, including proteome‑wide association studies (PWAS) and transcript-level analysis. In addition, we performed cell-type-specific expression to understand the expression patterns of specific proteins in different cell types and compared the consistency between the brain and blood. By constructing the protein-protein interaction (PPI) network, we further investigated the interactions among these proteins, and the interactions between candidate proteins and known drug targets. The goal of these integrative analyzes was to identify novel candidate risk protein markers and potential drug targets for SA.

## Methods

### Overall study design

**Fig. 1:**
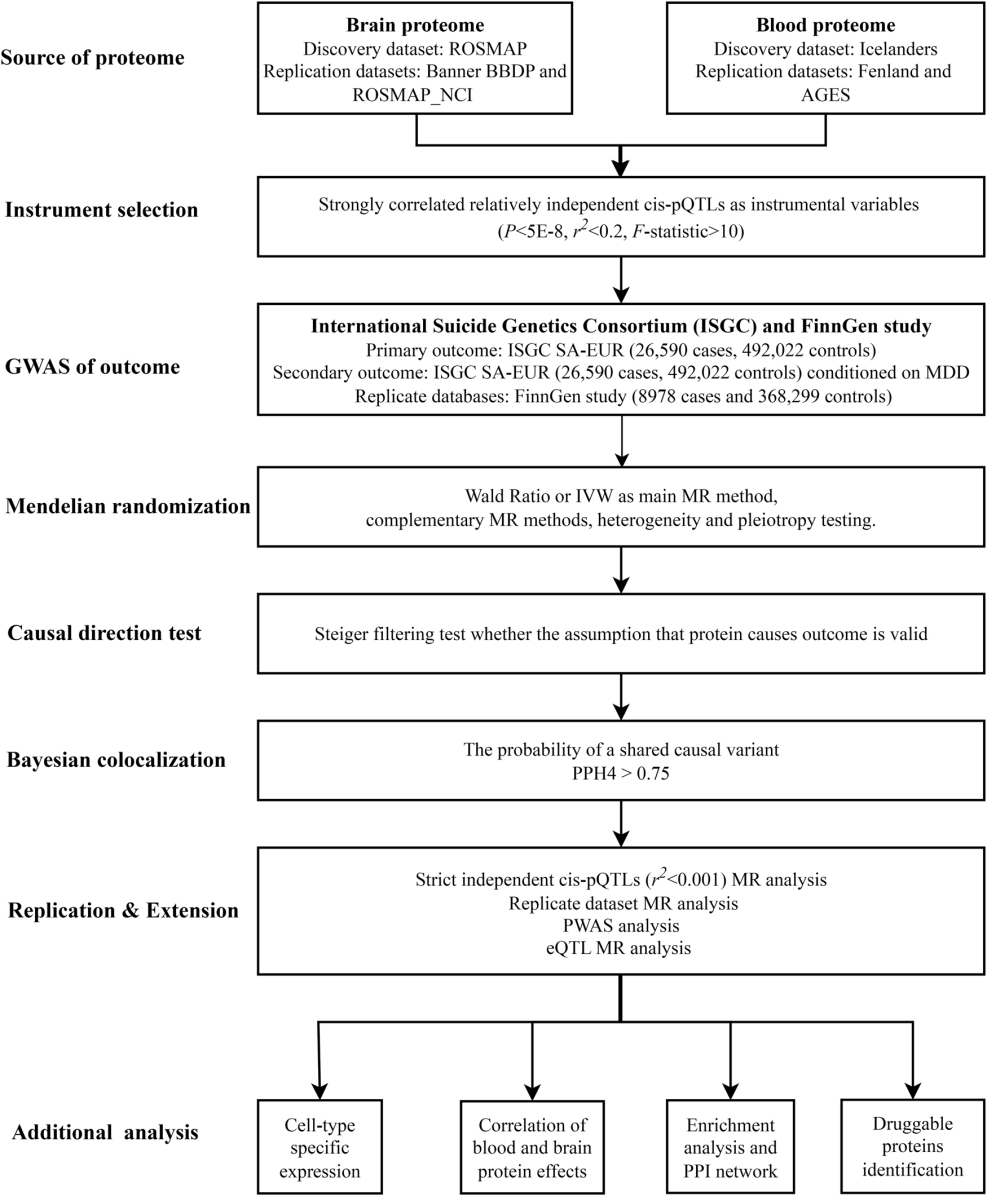
Schematic overview and framework of the present study design. First, we selected relatively independent cis-pQTLs from the brain and blood proteome datasets as IVs. Second, candidate proteins associated with SA were identified by a series of MR analyses. Third, the direction of causal association between candidate proteins and SA was ensured by Steiger filtering analysis. Fourth, whether proteins and SA share common causal variants was investigated by Bayesian colocalization. Fifth, to ensure the reliability of our findings, we conducted strictly independent cis-pQTL MR analysis, replicate dataset MR analysis, PWAS analysis, and eQTL MR analysis. Lastly, additional analyses encompassed cell-type-specific expression, correlation analysis between blood and brain protein effects, enrichment analysis, PPI network analysis, and identification of druggable proteins. ROS/MAP Religious Orders Study/Memory and Aging Project, Banner BBDP Banner Sun Health Brain and Body Donation Program, NCI normal healthy control, pQTL protein quantitative trait loci, SA suicide attempt, GWAS Genome-wide association studies, IVW inverse-variance weighted, MR Mendelian randomization, PWAS proteome‑wide association studies, eQTL expression quantitative trait loci, PPI protein-protein interaction.

This study utilized publicly available summary-level data on blood and brain proteome as well as GWAS data on SA. The schematic overview and framework of the present study design is shown in Fig. 1.

### Human brain proteome data source

For the discovery set, human brain proteome data were acquired from the dorsolateral prefrontal cortex (dPFC) of postmortem brain samples donated by 400 participants of European ancestry of the Religious Orders Study/Memory and Aging Project (ROS/MAP), which was the largest summary-level data on brain proteomes currently publicly available [22]. Proteomic sequencing and peptide analysis were performed using isobaric tandem mass tag peptide labeling and liquid chromatography coupled to mass spectrometry, respectively. Genotyping was performed from either the Illumina OmniQuad Express or Affymetrix GeneChip 6.0 platforms. The detailed method can be described in Wingo et al. [9]. After quality control, 376 subjects had both proteomic and genetic data for subsequent analysis.

Considering that the full discovery dataset included cognitively impaired participants, we used 144 participants with no cognitive impairment at death from the ROS/MAP for replication [8]. Another brain proteome data derived from dPFC of 149 individuals with both proteomic and genetic data from the Banner Sun Health Brain and Body Donation Program (Banner BBDP) were used for cross-study replication [23]. The proteomic data in the Banner BBDP were profiled using similar approaches to ROS/MAP.

### Human blood proteome data source

During the discovery phase, human blood proteome data were obtained from 35,559 Icelanders from the Icelandic Cancer Project and various genetic programs at deCODE genetics, Reykjavík, Iceland [7]. All plasma samples were measured using the SomaScan version 4 assay. Aptamers for non-human proteins and aptamers listed as deprecated by SomaScan as well as aptamers mapping to multiple genes were excluded, leaving 4907 aptamers targeted a total of 4719 unique proteins that were included in the pQTL analysis. Genome-wide association tests were performed using the linear mixed model implemented in BOLT-LMM by normalizing the residuals using model normal transformation and using normalized values as phenotypes. Detailed information can be found in the original study [7].

The human blood proteome measured from other sources was replicated separately: (1) Pietzner et al. conducted a study in which they detected 4775 unique protein targets from a cohort of 10,708 participants in the Finnish study [24]. (2) Gudjonsson et al. conducted a study in which they measured 4135 proteins from a cohort of 5368 individuals of Northern European ancestry participating in the AGES-Reykjavik Study [25].

### GWAS summary statistics of suicide attempt

For the primary analysis, summary statistics on SA were obtained from 14 cohorts of European ancestry (SA-EUR: 26,590 cases and 492,022 controls) from the International Suicide Genetics Consortium (ISGC) [5]. Cases were individuals who developed a non-fatal SA (13 cohorts) or died by suicide (1 cohort). A non-fatal SA was defined as a lifetime act of deliberate self-harm with intent to die. Information on SA was determined by structured clinical interviews in 8 cohorts, self-report questionnaires in 3 cohorts, hospital records or International Classification of Diseases codes in 2 cohorts. GWAS approach involved conducting analyses within European ancestry while accounting for confounding factors such as ancestry-informative principal components, genomic relatedness matrices, and factors capturing site of recruitment or genotyping batch as necessary. Detailed characteristics of these 14 European ancestry cohorts are shown in Table S1 and previous studies [5]. For external validation, summary statistics on SA were obtained from the FinnGen study (8978 cases and 368,299 controls) [26].

Considering that the major depressive disorder (MDD) is the most common psychiatric disorder among individuals who die by suicide and has the highest genetic association with SA [27], GWAS summary statistics for SA conditional on MDD (SA-EUR | MDD: 26,590 cases and 492,022 controls) were obtained based on multi-trait conditional and joint analysis (mtCOJO) for external validation [5]. Briefly, the mtCOJO adjusted the GWAS summary statistics of MDD for the effects of genetically related traits to determine putative SA-specific SNP associations [28]. Specifically, the effect size of SNPs on SA under the MDD condition was estimated.

### Statistical analysis

#### MR analysis

MR analysis was performed with brain and blood pQTLs as eligible IVs to infer the causal relationship between specific proteins and SA. Before MR analysis, IVs must meet three key assumptions: strongly associated with exposure, independent of confounders, or affecting outcome only through exposure [14]. To meet assumption 1, we first selected SNPs that achieved genome-wide association (*p* < 5E−08) and *F*-statistics >10, and then obtained conditionally independent SNPs (*r*^2^ < 0.2 and clump window >10,000 kb) as IVs through LD based on the 1000 Genomes European reference panel in subsequent analysis. Relatively relaxed clustering thresholds can improve the ability to detect effects and make it possible to test for bias in MR estimates [29]. To meet assumption 2 and 3, the following steps were taken further: (1) To minimize the risk of false positives, our study focused exclusively on cis-pQTLs, which are located within a 10 Mb range upstream and downstream of protein-coding genes. This approach was taken because trans-pQTLs, which map to genes not directly involved in coding for the targeted proteins or intergenic regions, make it difficult to ascertain the presence of vertical or horizontal pleiotropic pathways. (2) To avoid potential pleiotropic effects, SNPs of IVs that showed significant associations (*p* < 5E−08) with more than five proteins simultaneously were excluded from subsequent analysis. (3) To assess potential confounding effects, SNPs of each candidate protein were searched in the PhenoScanner database to determine whether they were significantly associated with potential confounding (*p* < 5E−08) [30]. (4) To avoid ambiguity or potential biases, SNPs with ambiguous palindromic structure were removed when exposure and outcome data were harmonized.

The primary MR analysis was performed using the Wald ratio (number of SNPs for IVs = 1) or the inverse variance weighted (IVW) method (number of SNPs for IVs >1) via the R-based “TwoSampleMR” package. The IVW method included fixed effects models and random effects models, depending on the presence of significant heterogeneity as assessed by Cochrane’s *Q* test [31]. To ensure the robustness of the results, complementary MR analysis methods can be implemented depending on the number of SNPs, including maximum likelihood ratio, MR-Egger, weighted median, and penalized weighted median analysis. The MR Egger intercept test further conducted to examine horizontal pleiotropy [32]. Finally, the Steiger filtering method was applied to ensure that causality was not distorted by the presence of reverse causality [15]. Multiple testing corrections were applied using false discovery rate (FDR) method with the “fdrtool” package considering the number of proteins analyzed [33]. A significance threshold of FDR < 0.05 was used to determine statistical significance, while findings with *p* < 0.001 that did not meet the FDR < 0.05 threshold were considered suggestive significant. This approach helps control for the possibility of false positives and ensures more reliable and stringent statistical interpretation of the results.

#### Bayesian colocalization analysis

Bayesian colocalization was performed to enhance the evidence of causality by assessing the posterior probability that the protein and SA share the same causal variant (rather than the variant being shared coincidentally due to correlation through LD) [16]. The colocalization method with default parameters tested the posterior probability of 5 hypotheses: H0: no association with either trait; H1: association with trait 1 (pQTL), not with trait 2 (SA GWAS); H2: association with trait 2 (SA GWAS), not with trait 1 (pQTL); H3: association with trait 1 (pQTL) and trait 2 (SA GWAS), with distinct causal variants; H4: association with trait 1 (pQTL) and trait 2 (SA GWAS), with a shared causal variant. We define genes based on the posterior probability PPH4 > 75% with strong evidence of colocalization [34].

#### Replication and extension analysis

To ensure the robustness of our results, we performed strictly independent cis-pQTL (*r*^2^ < 0.001) MR analysis. The independent cis-pQTL analysis allowed us to examine the causal associations between proteins and SA while minimizing potential confounding due to LD [29]. In addition, we performed MR analyses using replicate datasets, which involved using summary data for other protein sources and outcomes from SA-EUR|MDD and FinnGen study to validate our findings, ensuring the consistency and reliability of our results. Moreover, FUSION is a powerful strategy that combines protein abundance measurements with summary statistics from GWAS to identify genes whose cis-regulated protein abundance correlates with complex traits [35]. To further validate the association of candidate proteins with SA, PWAS were carried out using FUSION. Briefly, we used FUSION pipeline with default settings to combine the genetic effect of SA (SA GWAS *z*-score) with the protein weights by calculating the linear sum of *z*-score × weight for the independent SNPs at the locus to perform the PWAS of SA [35]. Furthermore, we performed additional validation by conducting MR analysis based on brain eQTLs at the transcriptional level using eQTL data obtained from the PsychENCODE project [36].

#### Cell-type-specific expression and correlation analysis

The cell-type-specific expression profile of genes associated with potential causal proteins in the brain was investigated using human single-cell RNA-seq data obtained from the Cell Types Database (https://portal.brain-map.org/atlases-and-data/rnaseq). Individual layers of the cortex were dissected, and nuclei were dissociated and sorted using the neuronal marker NeuN from human brain tissues. Nuclei were sampled from post-mortem and neurosurgical (MTG only) donor brains and expression was profiled with SMART-Seq v4 RNA-sequencing. To capture various aspects of expression specificity (ES), the cell-type expression specificity (CELLEX) tool was employed [37]. In addition, the correlation between the shared pQTLs identified in the brain and blood using effect estimates from the MR analysis was investigated by Pearson correlation analysis, and the different *p* value thresholds to investigate whether the correlations change as the significance increases.

#### Enrichment, PPI network, and druggable analysis

The Gene ontology (GO) and Kyoto Encyclopedia of Genes and Genomes (KEGG) pathway enrichment analysis was conducted to identify the GO terms and KEGG pathways enriched by the candidate protein [38]. To explore the interactions between SA-associated proteins identified in this study and investigate whether proteins identified using blood data could interact with proteins identified using brain data, we utilized the STRING database to investigate the PPI network for proteins with *p* < 0.05 based on MR analysis [39]. In addition, to assess the potential of identified proteins as therapeutic targets, we searched the Drug-Gene Interaction Database (https://www.dgidb.org/), DrugBank (https://www.drugbank.ca), and Open Targets (https://www.opentargets.org/) [40, 41] and explored the associations between identified proteins and established targets of anti-suicidal or antidepressant medications through the STRING database.

## Results

### MR and colocalization in the brain proteome

**Fig. 2:**
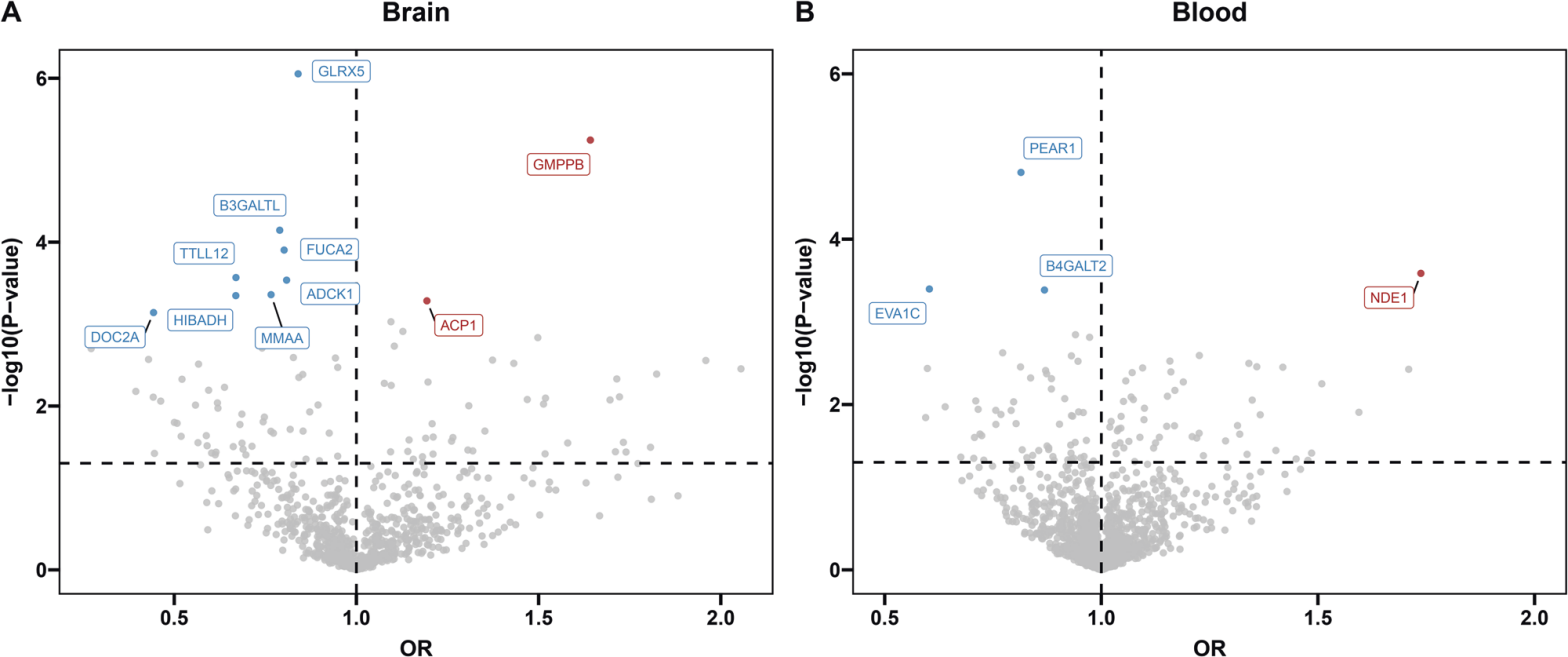
Volcano plots of the MR results for specific proteins on the risk of SA. **A** The MR results for 811 brain proteins on the risk of SA. **B** The MR results for 1333 blood proteins on the risk of SA. Odds ratios (OR) for increased risk of SA were expressed as per unit increase in protein levels. Red, blue, and gray dots denote positive significant, inverse significant, and nonsignificant associations, respectively. OR odds ratios, MR Mendelian randomization.

Primary MR analysis of brain pQTLs and SA GWAS revealed 94 proteins with genetically determined effects that showed suggestive evidence (*p* < 0.05; Tables S2 and S3). After multiple testing correction, 10 of these proteins remained significant (FDR < 0.05; Table 1 and Fig. 2A). Specifically, we observed that increased protein abundance of 8 proteins in the brain was significantly associated with a decreased SA risk, including glutaredoxin 5 (GLRX5; OR: 0.84; 95% CI: 0.78–0.90), beta 3-glucosyltransferase (B3GALTL; OR: 0.79; 95% CI: 0.70–0.89), alpha-L-fucosidase 2 (FUCA2; OR: 0.80; 95% CI: 0.72–0.90), tubulin tyrosine ligase like 12 (TTLL12; OR: 0.67; 95% CI: 0.54–0.83), AarF domain containing kinase 1 (ADCK1; OR: 0.81; 95% CI: 0.72–0.91), Metabolism of cobalamin associated A (MMAA; OR: 0.77; 95% CI: 0.66–0.89), 3-hydroxyisobutyrate dehydrogenase (HIBADH; OR: 0.67; 95% CI: 0.53–0.84), and double C2 domain alpha (DOC2A; OR: 0.44; 95% CI: 0.28–0.71), while increased protein abundance of 2 proteins in the brain was significantly associated with an increased SA risk, including GDP-mannose pyrophosphorylase B (GMPPB; OR: 1.64; 95% CI: 1.33–2.03) and Acid phosphatase 1 (ACP1; OR: 1.19; 95% CI: 1.08–1.32). Similar findings were found when applying robust MR approaches, including maximum likelihood, weighted median and penalized weighted median methods (Table S4). Cochrane’s *Q* test and MR Egger intercept test provided no evidence of heterogeneity or pleiotropy for the aforementioned proteins. The Steiger filtering analysis confirmed the correct causal direction from the protein level to SA for all MR-identified proteins (Table S5). However, Bayesian colocalization analysis revealed strong evidence of genetic colocalization with SA for only three out of the ten proteins, namely GLRX5, GMPPB, and FUCA2 (Table S6). This finding further supported their potential causal relationship with SA.

**Table 1:** Candidate proteins for SA identified by MR, Steiger filtering, and Bayesian colocalization analysis.

Tissue	Protein	Main MR analysis					Cochran’s *Q* *p* value	MR Egger intercept *p* value	Steiger filtering		Bayesian colocalization		Evidence of causal association
		Method	nSNPs	OR (95% CI)	*p* value	FDR			*p* value	Direction	PPH4 (%)	Colocalization	
Brain	GLRX5	IVW (fixed effects)	4	0.84 (0.78,0.9)	8.83E−07	0.001	0.930	0.705	1.00E−400	Correct	88.14	Yes	Yes
Brain	GMPPB	IVW (fixed effects)	2	1.64 (1.33,2.03)	5.68E−06	0.002	0.517	–	1.06E−34	Correct	75.45	Yes	Yes
Brain	B3GALTL	IVW (fixed effects)	2	0.79 (0.7,0.89)	7.16E−05	0.015	0.856	–	2.46E−62	Correct	24.04	No	No
Brain	FUCA2	Wald ratio	1	0.8 (0.72,0.9)	1.25E−04	0.021	–	–	1.11E−18	Correct	88.70	Yes	Yes
Brain	TTLL12	IVW (fixed effects)	2	0.67 (0.54,0.83)	2.71E−04	0.031	0.747	–	6.60E−38	Correct	14.85	No	No
Brain	ADCK1	IVW (fixed effects)	4	0.81 (0.72,0.91)	2.91E−04	0.032	0.750	0.531	5.00E−99	Correct	2.64	No	No
Brain	MMAA	IVW (fixed effects)	2	0.77 (0.66,0.89)	4.38E−04	0.037	0.294	−	2.59E−50	Correct	19.27	No	No
Brain	HIBADH	IVW (fixed effects)	3	0.67 (0.53,0.84)	4.50E−04	0.037	0.136	0.419	1.65E−54	Correct	1.38	No	No
Brain	ACP1	IVW (fixed effects)	4	1.19 (1.08,1.32)	5.21E−04	0.039	0.329	0.464	2.72E−301	Correct	5.58	No	No
Brain	DOC2A	Wald ratio	1	0.44 (0.28,0.71)	7.24E−04	0.048	–	–	9.45E−10	Correct	61.22	No	No
Blood	PEAR1	IVW (fixed effects)	2	0.81 (0.74,0.89)	1.56E−05	0.019	0.327	–	5.28E−112	Correct	42.44	No	No
Blood	NDE1	Wald ratio	1	1.74 (1.29,2.34)	2.59E−04	0.113	–	–	1.00E−09	Correct	30.71	No	No
Blood	EVA1C	Wald ratio	1	0.6 (0.46,0.8)	4.01E−04	0.126	–	–	4.69E−11	Correct	57.82	No	No
Blood	B4GALT2	IVW (fixed effects)	3	0.87 (0.8,0.94)	4.12E−04	0.127	0.661	0.692	3.87E−118	Correct	27.43	No	No

*MR* Mendelian randomization, *nSNPs* number of single nucleotide polymorphisms, *FDR* false discovery rate.

### MR and colocalization in the blood proteome

Primary MR analysis of blood pQTLs and SA GWAS identified 109 proteins that showed suggestive evidence of genetically determined effects (*p* < 0.05; Tables S7 and S8), but only one protein survived after multiple testing correction (FDR < 0.05; Table 1 and Fig. 2B). Genetically determined higher levels of platelet endothelial aggregation receptor 1 (PEAR1) were found to be associated with a decreased risk of SA (OR: 0.81; 95% CI: 0.74–0.89). The results from maximum likelihood MR analysis for PEAR1 plasma protein were consistent with those from the IVW method, and no heterogeneity was detected (Table S4). The Steiger filtering analysis supported that the correct causal direction from protein level to the development of SA for PEAR1 plasma protein (Table S5). Considering that blood pQTL studies measure proteins using aptamer-based approaches, we verified whether MR results were confounded by aptamer-binding effects. The IVs of PEAR1 (rs4661012 and rs4661075) were not known missense variants and did not exhibit high LD with missense variants. Therefore, the IVs were not susceptible to aptamer-binding effects. However, the colocalization results of PEAR1 (PPH4 = 42.44%) suggested the identified association might be a product of LD, indicating a lack of strong evidence for a causal relationship between PEAR1 and SA.

When we relaxed the significance threshold to *p* < 0.001, the three additional proteins (NDE1, EVA1C, and B4GALT2) identified by the MR analysis did not provide strong evidence for a causal association with SA in the colocalization analysis (Table S6), taking into account that the higher number of proteins analyzed in the blood affected the number of proteins that survived multiple corrections.

### Replication and extension analysis

To further confirm the significant causal association of the three candidate brain proteins (GLRX5, GMPPB, and FUCA2) with SA, we conducted a series of replication analyses (Table 2). Firstly, the causal associations of these three proteins with SA remained significant when considering strict independent IVs (*r*^2^ < 0.001) for SA from the discovery protein dataset and SA-EUR GWAS (Table S9). Secondly, we replicated the MR analysis of the multiple corrected surviving proteins in the discovery dataset, and found that the direction of effect of the available proteins was consistent with the findings in the discovery dataset (Table S10). This demonstrated the robustness of our results, despite the lack of instrumental variables for two of the three candidate proteins (GMPPB and FUCA2) in the replicate dataset. Thirdly, the significant causal associations of the three candidate brain proteins with SA were confirmed after external validation using mtCOJO adjustment for MDD as a secondary outcome (Table S11). Finally, the associations for GLRX5 and GMPPB were replicated in the FinnGen study, and the association for FUCA2 was directionally consistent in replication analysis (Table 2).

**Table 2:** Results of the replication and extension analysis of the three candidate brain proteins with causal evidence for SA.

Tissue	Protein	Strictly independent SNPs (LD < 0.001)		Secondary outcome (SA-EUR|MDD GWAS)		Replicate outcome (FinnGen GWAS)		PWAS analysis		eQTL MR analysis	
		OR (95% CI)	OR (95% CI)	OR (95% CI)	*p* value	OR (95% CI)	*p* value	*Z*-score	*p* value	OR (95% CI)	*p* value
Brain	GLRX5	0.85 (0.78,0.92)	0.85 (0.78,0.92)	0.85 (0.79,0.91)	3.81E−06	0.85 (0.73,0.99)	3.64E−02	−3.49	4.83E−04	0.70 (0.58,0.85)	2.72E−04
Brain	GMPPB	1.57 (1.22,2.02)	1.57 (1.22,2.02)	1.46 (1.17,1.82)	6.53E−04	1.74 (1.17,2.87)	3.10E−02	3.67	2.39E−04	1.15 (1.09,1.22)	9.58E−07
Brain	FUCA2	0.80 (0.72,0.90)	0.80 (0.72,0.90)	0.79 (0.71,0.89)	9.05E−05	0.90 (0.72,1.12)	9.46E−01	−3.53	4.22E−04	–	–

*MR* Mendelian randomization, *SNPs* single nucleotide polymorphisms, *LD* linkage disequilibrium, *SA-EUR* suicide attempt-European, *MDD* major depressive disorder, *GWAS* genome-wide association studies, *PWAS* proteome‑wide association studies, *eQTL* expression quantitative trait loci.

In addition, through confirmatory PWAS, we found that all three candidate brain proteins showed significant associations with SA risk (GLRX5: *Z*-score = −3.49, *p* = 4.83E−04; GMPPB: *Z*-score = 3.67, *p* = 2.39E−04; FUCA2: *Z*-score = −3.53, *p* = 4.22E−04) (Table S12). At the perspective of transcription level, two of the three candidate genes had suitable IVs, and MR analysis showed consistent results between the transcription level and the protein level (GLRX5; OR: 0.7; 95% CI: 0.58–0.85; GMPPB; OR: 1.15; 95% CI: 1.09–1.22) (Table S13). Taken together, strong evidence supports a significant causal association of these three candidate brain proteins with SA.

### Cell-type-specific expression of the SA-related genes

We utilized human single-cell RNA-seq data to investigate the specific enrichment and expression patterns of the three candidate brain proteins in different brain cell types (Table S14). Our analysis revealed that GLRX5 was predominantly enriched in pericyte and glutamatergic, GMPPB was specifically expressed in glutamatergic neurons, and FUCA2 showed expression in microglia, pericyte and vascular leptomeningeal cells (VLMC). These findings provide insights into the cell-type specificity of these proteins within the brain.

### Consistency comparison in brain and blood

**Fig. 3:**
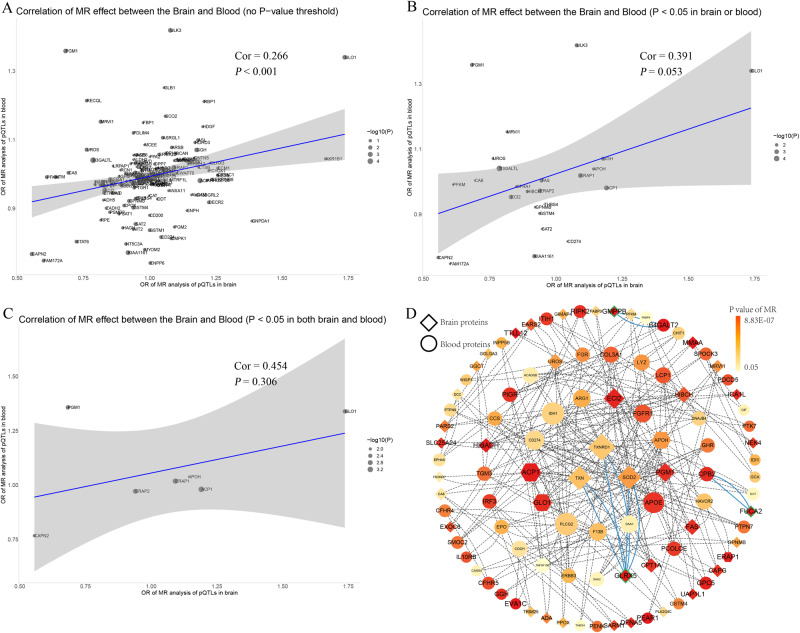
The correlation and PPI network between brain-based and plasma-based proteins. **A** Correlation of MR effect between brain and blood proteins (no *p* value threshold). **B** Correlation of MR effect between brain and blood proteins (*p* value < 0.05 in brain or blood). **C** Correlation of MR effect between brain and blood proteins (*p* value < 0.05 in both brain and blood). **D** PPI network for brain or blood proteins with *p* value < 0.05. MR Mendelian randomization, PPI protein-protein interaction.

To investigate the correlation between brain-based and plasma-based proteins, we compared the MR effect estimates for proteins that were shared between the two datasets. Firstly, we observed a mild correlation between the MR results for brain and blood proteins when considering all proteins without a specific *p* value threshold in the MR analysis (Pearson correlation = 0.266, *p* < 0.001, number of proteins = 151) (Fig. 3A). Secondly, when applying a *p* value threshold, the correlation coefficient increased to a moderate level, but it did not reach statistical significance (Fig. 3B, C). This could be attributed to the limited number of proteins that were shared between the brain and blood datasets (MR analysis with *p* < 0.05 in brain or blood: Pearson correlation = 0.391, *p* = 0.053, number of proteins = 25; MR analysis with *p* < 0.05 in both brain and blood: Pearson correlation = 0.454, *p* = 0.306, number of proteins = 7). These results suggested a potential correlation between the MR effect estimates of shared proteins, although larger sample sizes and more shared proteins were needed to establish statistical significance.

### Enrichment and PPI network, and potential drug targets

**Fig. 4:**
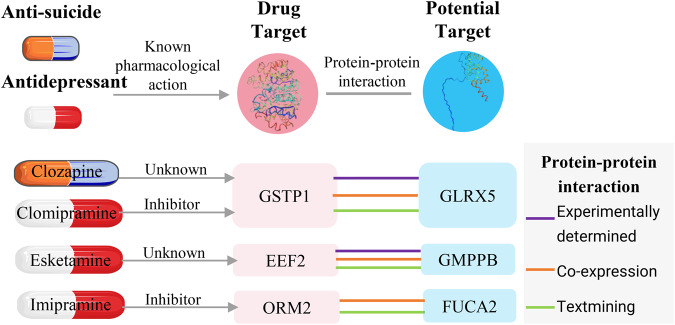
Interaction between current anti-suicidal or antidepressant medications targets and identified potential drug targets.

GO and KEGG enrichment analysis revealed that the three significant brain-based proteins were associated with various biological pathways, including metabolic pathways, lysosomes, neuronal cell bodies, and glutathione oxidoreductase activity (Table S15). PPI network analysis using the STRING database indicated potential interactions between the significant brain-based protein GLRX5 and the suggestive brain-based proteins TXN, TXNRD1, and SOD2 (Fig. 3D). In addition, we found protein interactions between important proteins from the brain and blood datasets. For instance, the significant brain-based protein GMPPB may be connected with the suggestive blood-based protein B4GALT2, while the significant brain-based protein FUCA2 may be connected with the suggestive blood-based protein CPB2. Moreover, STRING also revealed that GLRX5 was associated with Glutathione S-Transferase Pi 1 (GSTP1), the target of Clozapine and Clomipramine, GMPPB was associated with Eukaryotic Translation Elongation Factor 2 (EEF2), the target of Esketamine, and FUCA2 was associated with Orosomucoid 2 (ORM2), the target of Imipramine (Fig. 4).

## Discussion

In this study, we employed an integrated approach, including discovery and confirmatory study designs, integrating brain and blood-derived proteomic data with the largest SA GWAS data to date. Our aim was to investigate the genetically determined potential proteins associated with SA using comprehensive methods, including MR, Steiger filtering, colocalization, and PWAS. The results obtained from our analysis of brain proteomics data revealed the identification of 10 proteins (GLRX5, GMPPB, B3GALTL, FUCA2, TTLL12, ADCK1, MMAA, HIBADH, ACP1, DOC2A) that showed significant associations with SA. Notably, three of these proteins (GLRX5, GMPPB, and FUCA2) exhibited strong evidence of colocalization and were further replicated in in other datasets, providing robust support for their involvement in SA. In contrast, our analysis of blood proteomics data identified one protein with significant association (PEAR1) and three proteins with near-significant associations (NDE1, EVA1C, B4GALT2). However, these proteins lacked substantial evidence of colocalization, limiting the confidence in their role in SA. Furthermore, our findings highlight the significance of glutamatergic neuronal cells in the pathogenesis of SA, as the genes corresponding to brain proteins supported by MR evidence were predominantly expressed on the surface of these cells. In addition, despite the limited correlation between proteins in brain and blood, our analysis revealed certain proteins that participate in the same PPI network, suggesting potential interactions and crosstalk between brain and blood in the context of SA.

GLRX5 encodes a mitochondrial protein that plays a critical role in the biosynthesis of iron-sulfur clusters, which are required for maintaining normal iron homeostasis [42]. Several studies have indicated an association between blood iron homeostasis and SA risk [43], and abnormal iron homeostasis may be related to neurological and mental health [44, 45]. Nonetheless, there is a dearth of comprehensive research on the relationship between brain iron homeostasis and SA. Thus, our study provides a genetic basis for further determination of the need for comprehensive consideration of iron homeostasis in SA risk assessment and treatment. Moreover, we found that GLRX5 was predominantly enriched in pericytes and glutamatergic neurons. Pericytes play an important role in maintaining vascular function and the stability of the blood-brain barrier [46]. Glutamatergic neurons are one of the main types of excitatory neurons in the brain [47]. They play a key role in the transmission of neural information and the formation of neural networks by releasing glutamate as a neurotransmitter. Notably, the rapid antidepressant efficacy of ketamine is primarily based on the glutamate system [48]. In addition, the PPI network also revealed that GLRX5 interacts with GSTP1, the target of Clozapine—the first medication sanctioned by the US FDA for preventing suicidal behavior [4], suggesting its possible involvement in the mechanism of SA and its potential drug target value. In summary, our study not only provides valuable insights into the intricate relationship between iron homeostasis and SA but also offers a foundation for exploring the involvement of GLRX5 in blood-brain barrier stability and the functioning of glutamatergic neurons.

GMPPB encodes GDP-mannose pyrophosphorylase, which catalyzes the conversion of mannose-1-phosphate and GTP to GDP-mannose [49]. Previous studies have demonstrated dysregulation of GMPPB and mannose in depression, with increased levels of GMPPB proteins observed in the postmortem prefrontal cortex of patients with MDD and in a mouse model of chronic variable stress [50]. In addition, Deng et al. reported an association between elevated brain levels of GMPPB and an increased risk of MDD (OR [95%CI] = 1.452 [1.268–1.633]) by integrating proteomic data from the brain and MDD GWAS data [17]. Consistent with these findings, our study revealed that increased levels of GMPPB in the brain per standard deviation (SD) were associated with a 64% increased risk of SA. Notably, even after accounting for the effects of MDD, increased levels of GMPPB in the brain per SD remained associated with a 46% increased risk of SA. These results provide further support for the potential involvement of GMPPB in both MDD and SA. Moreover, the specific expression of GMPPB in glutamatergic neurons, and its interaction with EEF2—the target of Esketamine, a recently prominent novel antidepressant shown to alleviate suicidal ideation—further emphasizes its significance in the context of SA [51]. Our findings highlight the potential of GMPPB as a promising biomarker, however, further investigation and validation are necessary to elucidate the exact mechanism of action of GMPPB in SA.

FUCA2 encodes α-L-fucosidase and plays an important function in cellular lysosomes [52, 53]. Dysfunction of intracellular lysosomes has been linked to a variety of neurological diseases such as Parkinson’s disease and Alzheimer’s disease [54], as well as psychiatric disorders such as depression and SA [55]. The mechanisms underlying the association between lysosomal dysfunction and suicidal behavior are not fully understood, but several hypotheses may offer explanations. First, the accumulation of intracellular metabolites may lead to increased oxidative stress and cytotoxicity, resulting in damage to the nervous system [56]. This cellular damage may be involved in the development of SA. Second, lysosomal dysfunction may interfere with the synthesis, release, and clearance of neurotransmitters [57], thereby affecting the balance of the neurotransmitter system, which may adversely affect mood and cognitive functions, thereby increasing the risk of SA. In addition, FUCA2 also plays a regulatory role in the innate immune system and IGF transport. The innate immune system plays an important role in neurodevelopment, inflammatory responses, and neuronal protection [58]. As an important growth factor, IGF participates in key processes such as proliferation, growth and survival of nerve cells [59]. An in-depth understanding of the mechanism of action of FUCA2 in SA may help to develop new treatment approaches and prevention strategies, and provide a new direction for the intervention of SA.

Our study observed a significant but weak correlation between MR estimates of the same protein in brain and blood, although the strength of the correlation increased as we applied stricter *p* value thresholds in the MR analysis. This suggests that findings from one tissue are difficult to generalize directly to other tissues, especially when considering the effects of the blood-brain barrier [60]. This means that biomarkers between blood and brain may be tissue-specific, while their expression may differ in different tissues. Interestingly, our PPI network analysis revealed that some blood-based proteins extended the brain-based PPI network, such as GMPPB in the brain that may interact with B4GALT2 in the blood, FUCA2 in the brain that may interact with CPB2 in the blood. This suggests that brain state may influence certain peripheral biomarkers detected in SA, which are predominantly detectable in blood. On the other hand, the discovery of the PPI network may provide clues to further understanding the role of proteins detected in one tissue in the pathogenesis of SA in another. Although our evidence is still preliminary, our findings suggest that both the brain and blood may be valuable avenues for detecting proteins associated with SA. The three brain proteins we identified, along with their associated blood proteins, are promising candidates to be prioritized for future research. Further research can deeply explore the role of these proteins in the mechanism of SA, and provide more insights for the development of prediction and intervention strategies of SA.

Our research possesses several advantages. Firstly, we integrated pQTLs from brain and blood, using both a discovery and confirmatory study design. This comprehensive approach allowed us to thoroughly investigate and validate the key proteins implicated in the pathogenesis of SA. Secondly, we utilized the latest and largest GWAS dataset of SA, ensuring heightened statistical power and the reliability of our study. Additionally, our employed multiple independent yet complementary methodologies to identify novel SA-associated proteins, including MR analysis to uncover potential causal associations, Steiger filter analysis to ensure the correct direction of association, and Bayesian colocalization analysis to confirm that potential causal associations were not distorted by LD, PWAS to reinforce the evidence for brain proteins, and eQTL-based MR analysis to explore the association between the transcription levels of candidate brain proteins and SA. Lastly, we performed a comprehensive phenotypic GWAS scan of the candidate proteins identified in the MR analysis (Table S16), which showed that the IVs for these candidate proteins were not strongly correlated with other risks that may affect SA risk, suggesting that the candidate proteins are unlikely to exhibit widespread horizontal pleiotropy.

However, there are some limitations to our study that need to be considered. Firstly, the sample size of proteomic data obtained from brain tissue was small compared to the blood proteomic data, which limited the amount of brain proteins we were able to detect using pQTL. Secondly, our study focused only the dPFC region and blood samples, while other brain regions such as the hippocampus and amygdala have also been implicated in SA [61]. As protein data from different brain regions become more available in the future, it may provide more insights into the identification of proteins associated with specific brain regions. Thirdly, the brain pQTL datasets we used were from individuals with Alzheimer’s disease and cognitively normal older adults. Although we performed validation on cognitively normal people, past studies have shown that although genetic variants found to be associated with protein levels are not disease-specific nor age-specific [62]. However, a large sample of pQTL studies from healthy individuals of different ages may be more desirable. Fourthly, despite being the largest to date, the SA summary data from the ISGC faces limitations, notably in estimating SNP-heritability for SA at 7.5% based on SA-EUR, highlighting the challenge of capturing the complete genetic landscape. Additionally, data constraints hinder a comprehensive evaluation of various population, demographic, environmental factors, and confounding effects. Finally, our analysis was based on European samples, and further validation is necessary to assess the generalizability of our findings to individuals of non-European ancestry.

## Conclusions

In conclusion, our comprehensive analysis provides compelling evidence supporting a causal association of three genetically determined brain proteins (GLRX5, GMPPB, and FUCA2) with SA. These findings shed light on the involvement of glutamatergic neurons, iron homeostasis, and lysosomal dysfunction in the underlying mechanisms of SA. In addition, considering that the candidate potential protein interacts with established anti-suicide or antidepressant drug targets, making it a potential therapeutic target for suicide drug intervention, further animal experiments and clinical trials are needed in the future.

### Supplementary information

Supplemental Table 1-16

## Data Availability

Brain pQTL data from 376 subjects in the ROS/MAP study are available through 10.7303/syn23627957. The smaller pQTL data from 144 participants with no cognitive impairment in the ROS/MAP study are available through 10.7303/syn2580853. Brain pQTL data from the 149 participants in the Banner BBDP study are available through 10.7303/syn2580853. Blood pQTL data for all 4907 aptamers from 35,559 Icelanders are available at https://www.decode.com/summarydata/ and Supplementary Materials from the original study [7]. Blood pQTL data from 10,708 participants in the Finnish study are available in Supplementary Materials from the original study [24]. Blood pQTL data from 5368 participants in the AGES-Reykjavik Study are available in Supplementary Materials from the original study [25]. Data from the AGES-Reykjavik study are available through collaboration (AGES_data_request@hjarta.is) under a data usage agreement with the IHA. Brain eQTL data from the PsychENCODE are available in Supplementary Materials from the original study [36]. The International Suicide Genetics Consortium (ISGC) GWAS summary statistics for suicide attempt are available at https://tinyurl.com/ISGC2021. The FinnGen study GWAS summary statistics for suicide are available at https://www.finngen.fi/en.
